# Efficacy of 7‐benzyloxyindole and other halogenated indoles to inhibit *Candida albicans* biofilm and hyphal formation

**DOI:** 10.1111/1751-7915.13268

**Published:** 2018-04-15

**Authors:** Ranjith Kumar Manoharan, Jin‐Hyung Lee, Jintae Lee

**Affiliations:** ^1^ School of Chemical Engineering Yeungnam University Gyeongsan 38541 Korea

## Abstract

Certain pathogenic bacteria and yeast form biofilms on biotic and abiotic surfaces including medical devices and implants. Hence, the development of antibiofilm coating materials becomes relevant. The virulence of those colonizing pathogens can be reduced by inhibiting biofilm formation rather than killing pathogens using excessive amounts of antimicrobials, which is touted as one of the main reasons for the development of drug resistance. *Candida albicans* is an opportunistic fungal pathogen, and the transition of yeast cells to hyphal cells is believed to be a crucial virulence factor. Previous studies have shown that indole and its derivatives possess antivirulence properties against various bacterial pathogens. In this study, we used various indole derivatives to investigate biofilm‐inhibiting activity against *C. albicans*. Our study revealed that 7‐benzyloxyindole, 4‐fluoroindole and 5‐iodoindole effectively inhibited biofilm formation compared to the antifungal agent fluconazole. Particularly, 7‐benzyloxyindole at 0.02 mM (4.5 μg ml^−1^) significantly reduced *C. albicans* biofilm formation, but had no effect on planktonic cells, and this finding was confirmed by a 2,3‐bis‐(2‐methoxy‐4‐nitro‐5‐sulfophenyl)‐2H‐tetrazolium‐5‐carboxanilide (XTT) assay and three‐dimensional confocal laser scanning microscopy. Scanning electron microscopy analyses revealed that 7‐benzyloxyindole effectively inhibited hyphal formation, which explains biofilm inhibition. Transcriptomic analysis showed that 7‐benzyloxyindole downregulated the expressions of several hypha/biofilm‐related genes (*ALS3*,*ECE1*,*HWP1* and *RBT1*). A *C. albicans*‐infected *Caenorhabditis elegans* model system was used to confirm the antivirulence efficacy of 7‐benzyloxyindole.

## Introduction

Biofilms are microbial cells interwoven in an extracellular polymeric matrix that attach to abiotic and biotic surfaces. Pathogenic bacteria and fungi are protected by this three‐dimensional matrix, which confers them with high tolerance to antimicrobials. (Costerton *et al*., 1999; Davey and O'Toole G, 2000)*. Candida albicans* is an opportunistic fungal pathogen and causes systemic infections predominantly by contaminating implant devices such as pacemakers, endotracheal tubes, contact lenses, penile implants, intrauterine devices and catheters (Ramage *et al*., 2005; Sardi *et al*., 2013). *Candida albicans* biofilms contain cells in three development stages viz. yeast, pseudohyphae and hyphae. This colony dimorphism in *Candida* appears to regulate the maturation of biofilms and hyphal transition, the latter of which is considered a crucial virulence factor in *Candida* infections (Carradori *et al*., 2016). Hyphal formation in matured biofilms contains high densities of cells in a protected environment, which increases resistance to administered antimicrobials (Williams and Lewis, 2011). Owing to these properties, *C. albicans* biofilms are thought to be more strongly associated with the emergence of drug resistance than planktonic cells. Commercial antifungals for the treatment of candidiasis are limited to several azoles and polyenes (Tobudic *et al*., 2010; Taff *et al*., 2013; Sandai *et al*., 2016), and thus, small molecule novel antifungal agents are urgently required to prevent *C. albicans* biofilm formation.

Various studies have demonstrated that extracellular signalling molecules produced by bacteria can mediate quorum sensing (QS), and that QS molecules produced by one organism can modulate the community behaviour of host organisms as well as other organisms. These signalling molecules also direct the transcriptomic outcomes of bacterial genes associated with virulence and adaptive tolerance (Peleg *et al*. 2010). Several Gram‐positive and Gram‐negative bacteria synthesize indoles as intracellular signalling molecules to control the virulence of pathogenic bacteria, such as, *Pseudomonas aeruginosa* and enterohaemorrhagic *E. coli* O157:H7 (Lee *et al*., 2007, 2011). In *Pseudomonas putida*, signalling molecules such as indole enhances TtgGHI efflux pump that are relevant for antibiotic resistance (Molina‐Santiago *et al*., 2014). The previous studies have reported that indole inhibits biofilm formation and suppresses the virulence of bacterial strains such as *Staphylococcus aureus*,* Agrobacterium tumefaciens* (Lee and Lee, 2010; Lee *et al*., 2013, 2015a,b; Lee *et al*., 2016) and *Vibrio cholera* (Mueller *et al*., 2009). Likewise, indole derivatives such as 7‐fluoroindole, 7‐hydroxyindole, 3‐indolyl acetonitrile and 2‐aminobenzimidazoles have been reported to exhibit antimicrobial activities against pathogenic bacteria (Lee *et al*., 2009, 2011, 2012, 2015a,b; Frei *et al*., 2012).

Like bacteria, fungi such as *Aspergillus* sp. and *Penicillium* sp., produce indole derivatives that have been reported to inhibit *C. albicans* biofilm formation and hyphal development (Wang *et al*., 2012; You *et al*., 2013). Although relatively few reports are available to conclude, indole and indole‐3‐acetonitrile have been shown to suppress biofilm maturation by *C. albicans* (Jayant *et al*., 2012; Oh *et al*., 2012).

In this study, crystal violet and XTT (2,3‐bis(2‐methoxy‐4‐nitro‐5‐sulfo‐phenyl)‐2H‐tetrazolium‐5‐carboxanilide) reduction assays showed efficiency of 7‐benzyloxyindole on biofilm formation by *C. albicans*. Cell morphology and phenotypic switching of *C. albicans* biofilm cells were observed by scanning electron microscopy (SEM), and biofilm thicknesses were measured by confocal laser scanning microscopy (CLSM). In addition, transcriptomic studies were performed to determine the antibiofilm and antihyphal effects of 7‐benzyloxyindole in *C. albicans*. Finally, the effects of 7‐benzyloxyindole were investigated in a *C. albicans*‐infected *Caenorhabditis elegans* (a nematode) model.

## Results

### Effects of indole derivatives on *C. albicans* biofilm formation

**Figure 1:**
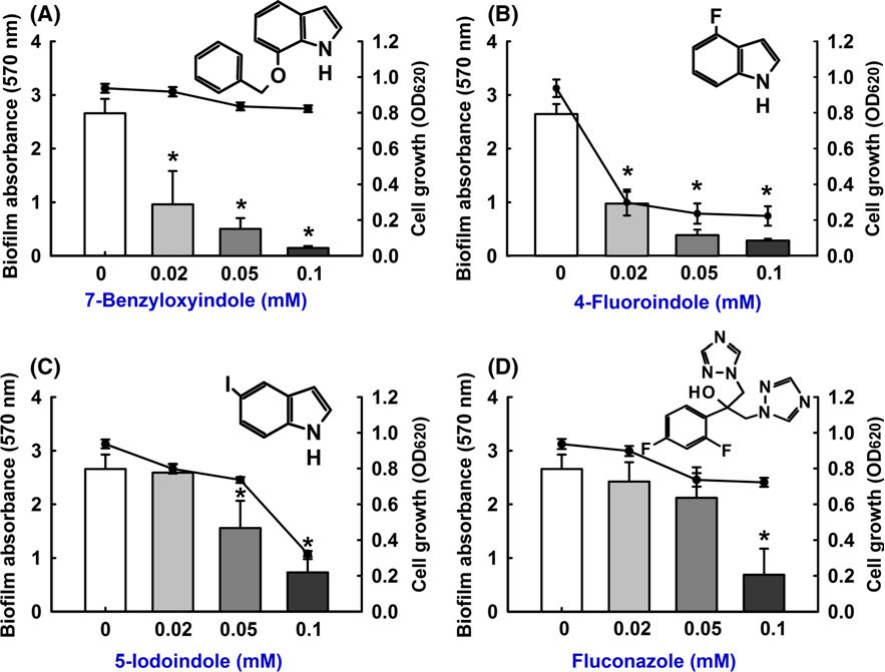
Inhibition of biofilm formation by indole derivatives. The antibiofilm activities of 7‐benzyloxyindole (A), 4‐fluoroindole (B), 5‐iodoindole (C) and fluconazole (D) against *C. albicans* DAY185 were determined after culturing for 24 h. Two independent experiments were conducted (six wells per sample); error bars indicate standard deviations. *P <* *0.05 vs. non‐treated controls. Bars indicate biofilm formation and lines indicate planktonic cell growth.

Initially, we investigate whether indole derivatives affect biofilm formation by fluconazole‐resistant *C. albicans* DAY185 (Manoharan *et al*., 2017a,b), and cell growth was simultaneously measured in the presence of indole derivatives. Of the 34 commercially available indole derivatives examined, 7‐benzyloxyindole, 4‐fluoroindole and 5‐iodoindole significantly reduced biofilm formation at concentrations of 0.1 and 0.5 mM (Table S1). In particular, 7‐benzyloxyindole and 4‐fluoroindole significantly inhibited biofilm formation in a dose‐dependent manner (Fig. 1). More specifically, 7‐benzyloxyindole significantly inhibited biofilm formation by 63%, 81% and 94% at concentrations of 0.02, 0.05 and 0.1 mM, respectively (Fig. 1A). The commercial antifungal fluconazole (positive control) significantly reduced biofilm formation by 74% at a concentration of 0.1 mM (Fig. 1D). In addition, 4‐fluoroindole and 5‐iodoindole demonstrated wide range inhibition of the growth of *C. albicans* at the planktonic cell stage (Figs. 1B and C). Interestingly, planktonic cell growth was not affected by 7‐benzyloxyindole at a concentration of 0.1 mM (Fig. 1A), and minimum inhibitory concentrations (MIC) exhibited up to 2 mM against *C. albicans*. Thus, confirming biofilm inhibition by 7‐benzyloxyindole was due to its antibiofilm activity rather than its antimicrobial activity.

### Effects of 7‐benzyloxyindole on *C. albicans* metabolic activity

**Figure 2:**
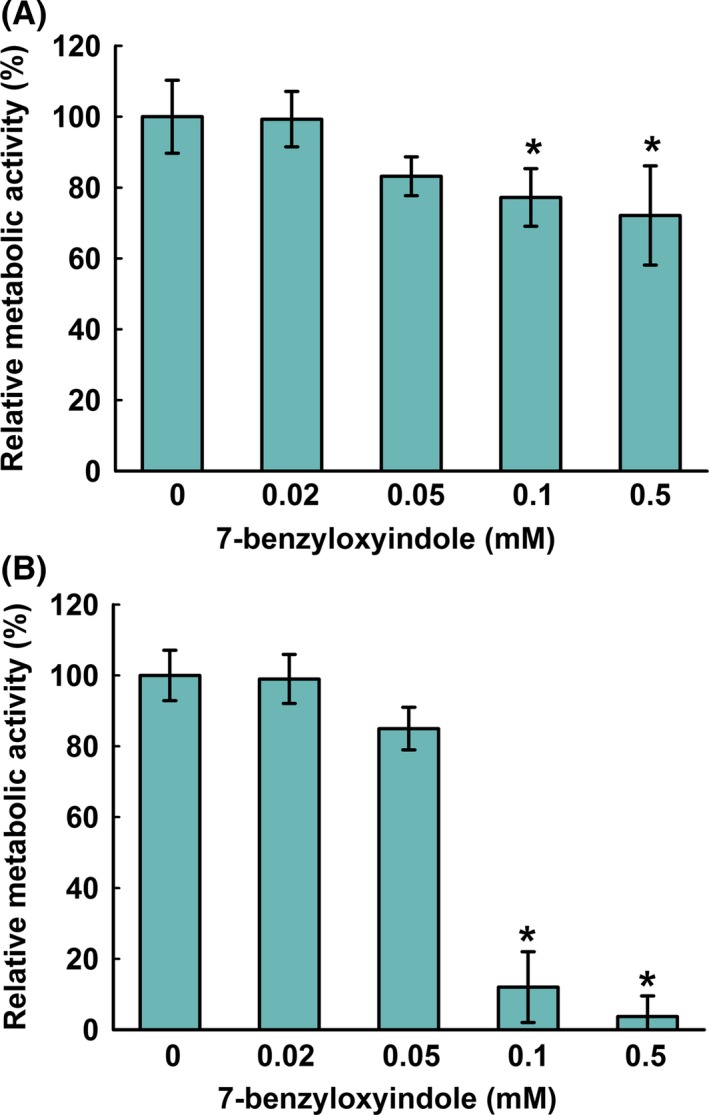
Metabolic activity of 7‐benzyloxyindole against *C. albicans*. The metabolic activities of *C. albicans* planktonic cells (A) and biofilms (B) were quantified using an XTT assay in the presence of 7‐benzyloxyindole after incubation for 24 h. Results are presented as mean percentages of metabolic activity versus non‐treated controls. Two independent experiments were conducted (six wells per sample); error bars indicate standard deviations. None indicates non‐treated samples. *P <* *0.05 vs. non‐treated controls.

Colorimetric assays are valuable for quantifying the viabilities of eukaryotic cells, and it has been suggested the XTT assay is useful to study fungal biofilm formation and drug resistance (Chandra *et al*., 2001; Kuhn *et al*., 2002). Findings from our XTT assay showed that metabolic activity of biofilm and planktonic *C. albicans* cells was not affected after 7‐benzyloxyindole treatment at 0.02 and 0.05 mM (Fig. 2). As expected, biofilm cell viabilities were significantly reduced by 88% and 96% by 7‐benzyloxyindole at 0.1 or 0.5 mM, respectively. However, planktonic cell viabilities were only slightly affected by 7‐benzyloxyindole at these concentrations (Fig. 2A).

### 7‐Benzyloxyindole affected *C. albicans* morphology

**Figure 3:**
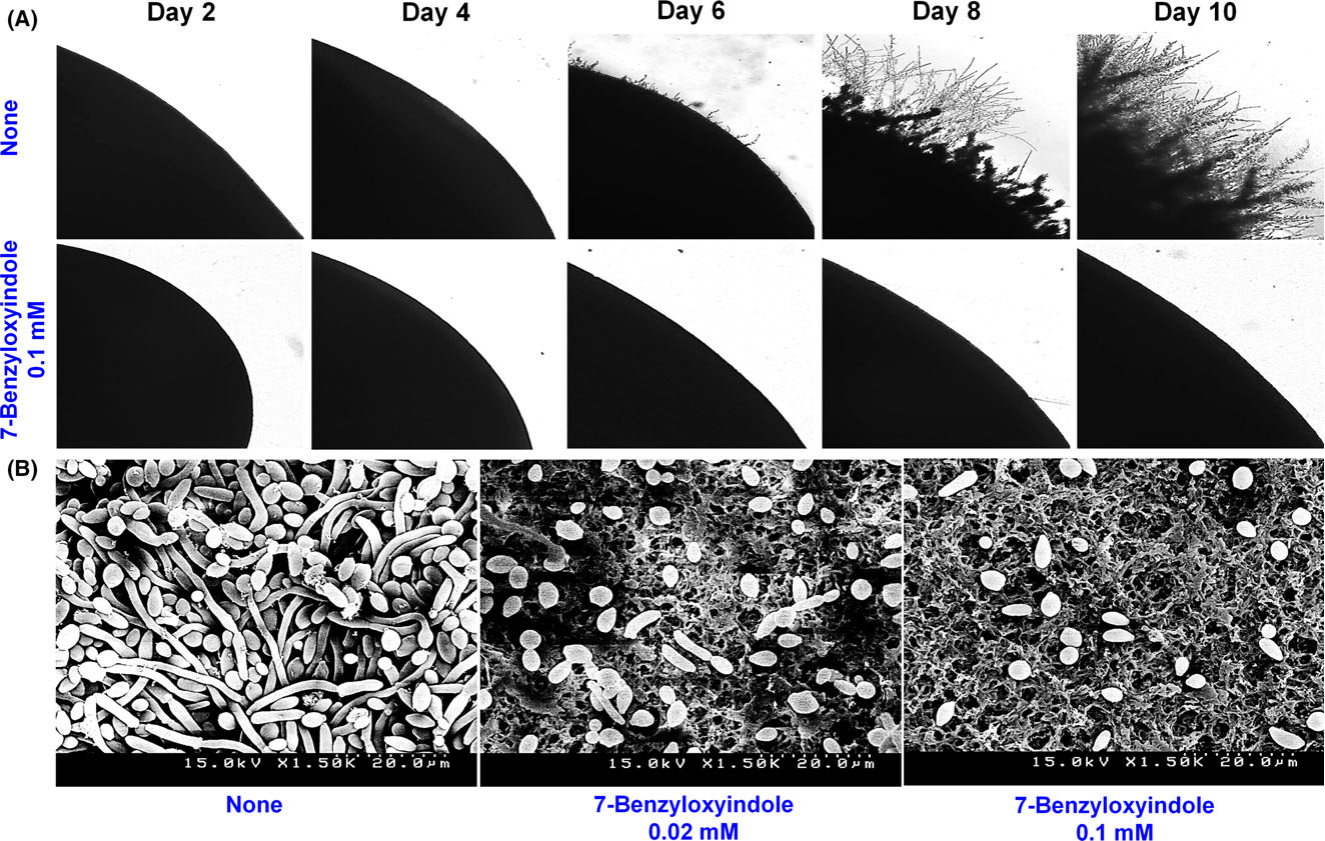
Effects of 7‐benzyloxyindole on *C. albicans* morphology. *C. albicans* morphology on solid media (A). *C. albicans* was streaked on PDA agar plates in the absence or presence of 7‐benzyloxyindole (0.1 mM). Colony morphology was photographed at every two days at 37°C. Inhibition of hyphal growth by 7‐benzyloxyindole was visualized by SEM (B). The scale bar represents 20 μm. At least two independent experiments were conducted. None indicates non‐treated control.

To examine the inhibitory effect of 7‐benzyloxyindole on *C. albicans* morphology, visual microscopy, SEM and CLSM were performed. Initially, the effect of 7‐benzyloxyindole on *C. albicans* hyphal growth on solid media was examined by cultivating fungal cell colony on PDA agar plate at 37°C. While filament formation on untreated colony was observed after 6 days of incubation, 0.1 mM of 7‐benzyloxyindole was adequate to inhibit filamentation for 10 days (Fig. 3A). Also, SEM analysis showed that 7‐benzyloxyindole was found to substantially suppress hyphal growth in biofilms at concentrations of 0.02 and 0.1 mM on nylon membranes (Fig. 3B), and at 0.02 mM inhibited hyphal cells, which led to an accumulation of pseudohyphae and yeast cells.

**Figure 4:**
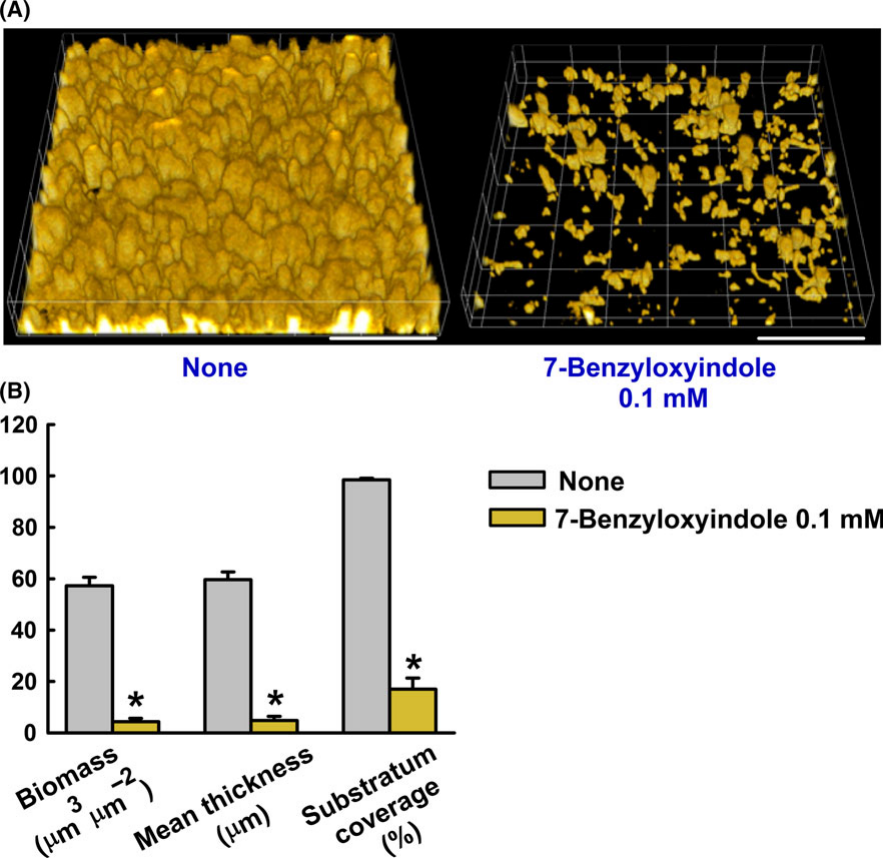
Microscopic observations of the effects of 7‐benzyloxyindole on biofilms. Biofilm formation by *C. albicans* on polystyrene plates was observed in the presence of 7‐benzyloxyindole at 0.1 mM by confocal laser microscopy (A). Scale bars represent 100 μm. Biofilm formation was quantified by COMSTAT (B) *P < 0.05 vs. non‐treated controls.

Confocal laser scanning microscopy analysis showed untreated *C. albicans* formed dense biofilms, and that 7‐benzyloxyindole at 0.1 mM dramatically reduced cellular densities and biofilm thicknesses (Fig. 4), in turn blocking biofilm formation as determined by crystal violet assays. Furthermore, COMSTAT analysis showed 7‐benzyloxyindole at 0.1 mM to significantly reduce biofilm biomass, average thickness and substrate coverage (Fig. 4B). More specifically, biofilm biomass and mean thickness after treatment were reduced by up to ≥ 90% versus untreated controls. Likewise, 7‐benzyloxyindole at 0.1 mM reduced substrate coverage by 82% (Fig. 4B). These results showed 7‐benzyloxyindole to effectively inhibit hyphal formation and biofilm maturation by *C. albicans* in the liquid medium and on solid plates. These findings suggest 7‐benzyloxyindole to probably downregulate the expression of genes that promote hyphal formation and biofilm maturation.

### Effect of 7‐benzyloxyindole on the expression of hypha‐specific and biofilm‐related genes

**Figure 5:**
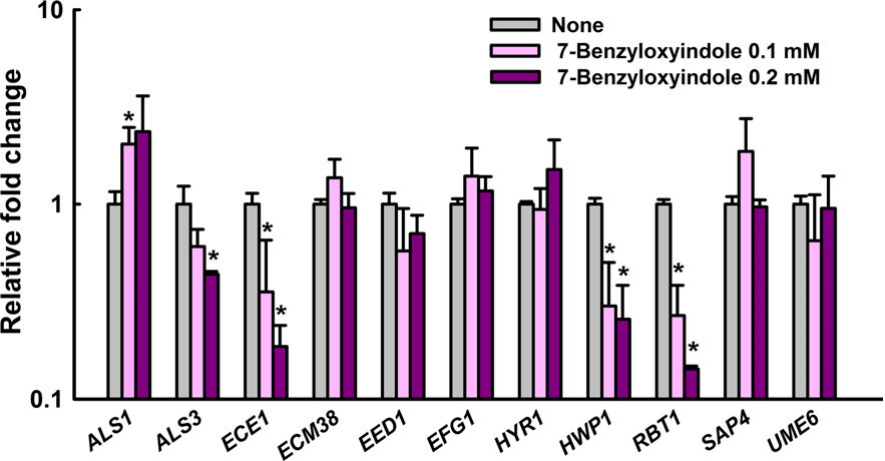
Transcriptional profiles of *C. albicans* cells treated with or without 7‐benzyloxyindole. *C. albicans* was cultivated with or without 7‐benzyloxyindole (0.1 mM and 0.2 mM) for 4 h. Transcriptional profiles were measured by qRT‐PCR. Relative expressions represent transcriptional levels after treatment with 7‐benzyloxyindole versus non‐treated controls. Fold changes represents transcription changes in treated *C. albicans* versus non‐treated controls. The experiment was performed in duplicate. Error bars indicate standard deviations. *P <* *0.05 vs. non‐treated controls.

Transcriptional levels of hypha‐specific and biofilm‐related genes in *C. albicans* were quantified by qRT‐PCR. We found 7‐benzyloxyindole at 0.1 mM to significantly reduce the mRNA levels of the hypha‐specific genes *HWP1* (3.3‐fold) and *RBT1* (3.8‐fold) versus respective non‐treated controls (Fig. 5). Also, *HWP1* (fourfold), *ALS3* (2.5‐fold), *RBT1* (7.1‐fold) and *ECE1* (5.5‐fold) levels were reduced significantly after treatment with 7‐benzyloxyindole at 0.2 mM. Interestingly, *ALS1,* which is involved in biofilm formation, was found to be upregulated by 7‐benzyloxyindole treatment, whereas transcription factor *EFG1* to be only slightly affected after the treatment (Fig. 5). Taken together, qRT‐PCR results showed that 7‐benzyloxyindole significantly altered the expression of some hypha‐specific and biofilm‐related genes.

### Efficacy of 7‐benzyloxyindole in the nematode *Caenorhabditis elegans*

**Figure 6:**
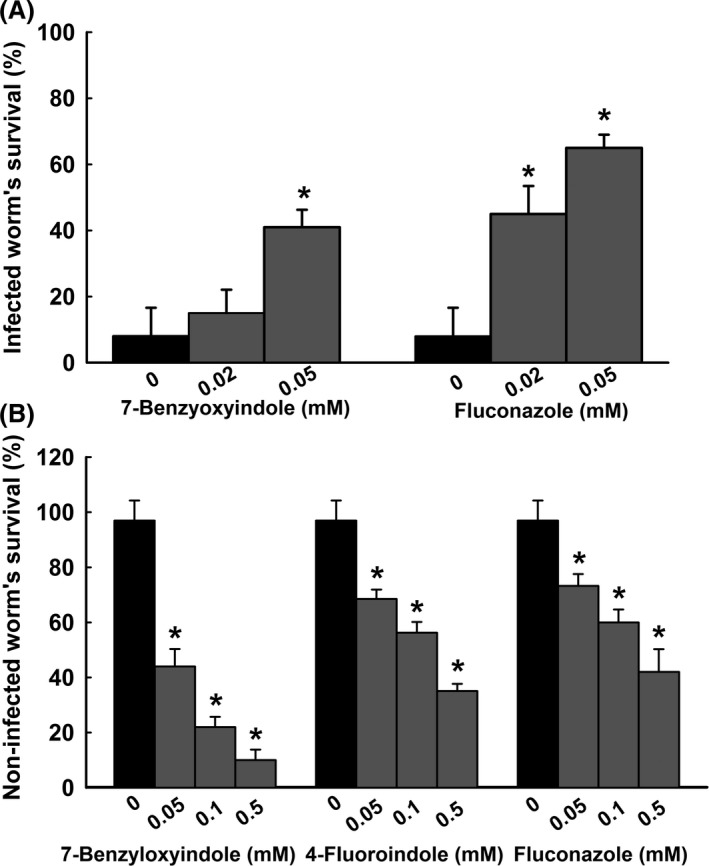
Effects of 7‐benzyloxyindole on *C. albicans*‐infected *C. elegans*. The bar graph indicates percentage worm survival after exposure of *C. albicans* for 4 days to 7‐benzyloxyindole. Fluconazole was used as a positive control (A). The toxicities of 7‐benzyloxyindole, 4‐fluoroindole and fluconazole were studied on non‐infected nematodes survival after 4 days (B). None indicates non‐treated controls. Worm survival was determined based on movement. At least two independent experiments were conducted. Error bars indicate standard deviations. *P <* *0.05 vs. non‐treated controls.

In this study, we examined whether 7‐benzyloxyindole could affect *Candida* virulence in a *Caenorhabditis elegans* nematode model –an alternative to mammalian models (Tampakakis *et al*., 2008). Microscopic observations of infected nematodes revealed that *C. albicans* infection caused 92% fatality in 4 days (Fig. 6A). However, more than 40% of nematodes survived 4 days in the presence of 7‐benzyloxyindole at 0.05 mM, and > 60% survived 4 days in the presence of fluconazole (a commercial antifungal agent) at same concentration (Fig. 6A). To study the toxicity of 7‐benzyloxyindole and 4‐fluoroindole, nematodes without *C. albicans* infection were exposed to these compounds for 4 days. It was found that 4‐fluoroindole exhibited mild toxicity which is similar to commercial antifungal agent fluconazole at same concentrations (Fig. 6B). Compared to 4‐fluoroindole and fluconazole, 7‐benzyloxyindole showed more toxicity to nematodes. For instance, 55% worms survived at 0.1 mM 4‐fluoroindole, whereas 22% worms survived at same concentration of 7‐benzyloxyindole (Fig. 6B).

## Discussion

Increase in the prevalences of multidrug‐resistant *Candida* strains has encouraged the investigations on the activities of small molecules that play important roles in the inhibition of biofilm formation. Consequently, we searched for new indole derivatives that inhibit biofilm formation rather than only cell growth to reduce the risk of drug‐resistance development. Previously, we have shown that 7‐benzyloxyindole had antivirulence activity and antibiofilm activity against persistent *S. aureus* infections *in vitro* (Lee *et al*., 2013). In the present study, we studied benzyloxy and fluoro‐substituted indoles, and found that benzyloxy group is essential for antibiofilm activity against the fluconazole‐resistant *Candida* strain (Fig. 1). It has been previously reported that benzyloxy derivatized compounds have beneficial immunological effects in human epithelial cells such as antisickling activity (Abraham *et al*., 1984; Mahran, 2000). Another study has shown that functional groups of indoles such as indole carboxamide derivatives better inhibited *C. albicans* biofilms than propenamide derivatives (Olgen *et al*., 2008).

The metabolic activity experiments showed that *C. albicans* cells were viable after 7‐benzyloxyindole treatment (Fig. 2), which is consistent with other reports that the small molecules such as indole are not toxic to *C. albicans* (Olgen *et al*., 2008; You *et al*., 2013). Altman (1976) reported mitochondrial succinoxidase, cytochrome P450 and flavoprotein oxidases are primarily responsible for the conversion of XTT to coloured formazan, which is important in fungal research because XTT conversion provides a measure of metabolic activity, which is related to fungal wall biosynthesis. (Kuhn *et al*., 2002). This findings imply that 7‐benzyloxyindole at concentrations of >0.1 mM reduced metabolic activity due to low levels of C. albicans cells in biofilms (Fig. 2B). The inhibited hyphal formation after 7‐benzyloxyindole treatment could be associated with shortening of hyphal length and accumulation of pseudohyphae, which may be due to phenotypic plasticity, as *C. albicans* has also been repeatedly shown to undergo morphological changes from the hyphal to yeast form in the presence of environmental stress (Lu *et al*., 2011; Vediyappan *et al*., 2013).

Here, 7‐benzyloxyindole treatment downregulated *HWP1*,* ALS3*,* ECE1* and *RBT1* hyphae‐specific and biofilm‐related genes (Fig. 5). It has been reported *HWP1* and *ALS3* mutants are defective in terms of *C. albicans* biofilm development (Nobile *et al*., 2006a,b). Hyphal formation by *C. albicans* is regulated by the Ras‐cAMP‐Efg1 signalling pathway. In detail, small GTPase *RAS1* activates cAMP, which promotes the PKA‐mediated activation of transcription factor *EFG1*, which in turn regulates hyphae‐specific genes, such as *ALS3*,* HWP1*, and *ECE1*, and thus, modulates hyphal formation (Leberer *et al*., 2001). *RBT1* (repressed by *TUP1*) encodes cell surface proteins that are regulated by Tup1, which exhibits high similarity to *HWP1* (Braun *et al*., 2001). Here, we suggest 7‐benzyloxyindole inhibits biofilm formation by modulating Ras‐cAMP‐Efg1 signalling pathway genes (*HWP1*,* RBT1* and *ECE1*), which are strongly associated with long‐term hyphal maintenance, and thus, reducing hyphal development.

Previously, it was shown that hyphal form of *C. albicans* kills by piercing nematode cuticles, and that the yeast form is non‐lethal (Pukkila‐Worley *et al*., 2009). Similarly, it was reported that the survival rates of *C. albicans*‐infected nematodes were increased by treatment with gymnemic acid (Vediyappan *et al*., 2013), retigeric acid (Chang *et al*., 2012) or polyphenolic compounds such as magnolol and honokiol (Sun *et al*., 2015). This implies that 7‐benzyloxyindole could rescue the animals from *Candida* infection by preventing yeast‐hyphal transition (Fig. 6A). The correlations for toxicities between *C. elegans* and rodents make the case of inclusion of *C. elegans* for toxicity assessment (Dengg and van Meel, 2004; Sochova *et al*., 2006). Our results suggest that tested compounds could use for hyphal inhibition in animals with low dosage. Consistent with previous reports (Berman and Sudbery, 2002; Saville *et al*., 2003), our study of the indole compounds that we chose led us to speculate that they may be effective against invasive hyphae formation in patients with candidiasis.

In conclusion, the present study indicates indole derivatives such as 7‐benzyloxyindole could be used to control fungal virulence by regulating hyphae‐specific genes and to treat biofilm‐associated infections on medical implant devices and *Candidiasis* infections.

## Experimental procedures

### Strains and medium

In this study, *C. albicans* strain DAY185 was cultured in potato dextrose agar (PDA) and preserved in 1 ml of potato dextrose broth (PDB) supplemented with 30% glycerol at –80°C until use. As previously reported, DAY185 is resistant to the commercial antifungal fluconazole (MIC ~ 512 μg ml^−1^) (Manoharan *et al*., 2017a,b). A single colony was inoculated into 25 ml of PDB and incubated for overnight at 37°C. All compounds tested for this study were purchased from Sigma‐Aldrich (St. Louis, MO, USA) and Combi Blocks, Inc. (San Diego, CA, USA) and were dissolved in dimethyl sulfoxide (DMSO), which did not exceed 0.1% (vol/vol) in any experiment. The cell growths and turbidities were measured using spectrophotometer (UV‐160, Shimadzu, Japan) at 620 nm. Overnight *C. albicans* cells were prepared at the density of 10^5^ CFU ml^−1^ with the presence of tested compounds in 96‐well polystyrene plates (SPL Life Sciences, Pocheon, Korea) to determine MIC using the Clinical Laboratory Standards Institute dilution method with slight modification (Alastruey‐Izquierdo *et al*., 2015). The plates were then incubated for 24 h at 37°C and the lowest concentration that inhibited yeast growth by at least 80%, as assessed by spectrophotometry (620 nm) and colony counting was determined as MIC.

### Assays for biofilm formation

*Candida* biofilms were developed on 96‐well polystyrene plates, as previously reported (Lee *et al*., 2011). Briefly, *C. albicans* overnight cultures at an initial turbidity of 0.1 at 600 nm were inoculated into PDB (final volume 300 μl) with or without test compounds at varying concentrations and incubated for 24 h without shaking at 37°C. To determine biofilm formation, non‐adherent cells were removed by washing plates three times with H_2_O, crystal violet staining for 20 min followed by washing three times, and extracting the crystal violet using 95% ethanol. The results were presented as bar graphs as the average of at least six replicates by measuring absorbance at 570 nm.

### Biofilm metabolic activity –XTT reduction assay

Biofilm growth was analysed using a XTT [2,3‐bis(2‐methoxy‐4‐nitro‐5‐sulfophenyl)‐2H‐tetrazolium‐5‐carboxanilide sodium salt] reduction assay using established procedures (Ramage *et al*., 2001; Nett *et al*., 2011). 300 μl of cell suspension diluted 10^5^ CFU ml^−1^ was inoculated into PDB with or without 7‐benzyloxyindole at different concentrations for 24 h without shaking at 37°C. The metabolic activities of biofilm cells were measured by mixing freshly prepared XTT and menadione solutions (Sigma‐Aldrich) at 20∶1 (v/v). To each well, XTT‐menadione solution (42 μl) and PBS (158 μl) were added to prewashed biofilms, and incubated at 37°C in the dark for 3 h. The obtained coloured supernatant (100 μl) was transferred to new microtiter plates, and measured by absorbances at 450 nm. Similarly, planktonic cell viability was measured using culture supernatants.

### Colony morphology of *C. albicans* on solid media

A freshly prepared glycerol stock of *C. albicans* was used to streak on PDA agar plates supplemented with DMSO or 0.1 mM concentration of 7‐benzyloxyindole. The plates were then incubated for 10 days at 37°C, and the temporal colony morphology was photographed at every alternate day using an iRiS^™^ Digital Cell Imaging System (Logos Bio Systems, Anyang, Korea).

### Observations of hyphae by scanning electron microscopy (SEM)

Hyphal formation of *C. albicans* was observed by SEM, as previously described (Lee *et al*., 2014). Small pieces (0.5 × 0.5 cm) of nylon filter were placed in each well of 96‐well plates containing 200 μl cells suspension/well at the density of 10^5^ CFU ml^−1^. Cells were incubated in the absence (untreated) or presence of 7‐benzyloxyindole at 37°C for 24 h without shaking, fixed with glutaraldehyde (concentration 2.5%) and formaldehyde (concentration 2%) for 24 h, and serially postfixed using sodium phosphate buffer and osmium tetroxide, dehydrated using an ethanol series (50, 70, 80, 90, 95 and 100%), and isoamyl acetate. After critical‐point drying, cells on nylon filter were examined under an S‐4200 scanning electron microscope (Hitachi, Tokyo, Japan) at magnifications ranging from × 2000 to ×10 000 and an accelerating voltage of 15 kV.

### Confocal laser scanning microscopy of biofilm formation

*Candida albicans* biofilms were grown in 96‐well plates in the absence or presence of 7‐benzyloxyindole (0.1 mM) without shaking. Planktonic cells were then removed by washing with H_2_O three times. Carboxyfluorescein diacetate succinimidyl ester (a minimally fluorescent lipophile; Catalog #: C34554; Invitrogen, Molecular Probes, Inc, Eugene, OR, USA)(Weston and Parish, 1990) was used to stain *C. albicans* cells. The bottom of 96‐well plates was visualized using an (a 488 nm) Ar laser (emission wavelength 500 to 550 nm) under a confocal laser microscope (Nikon Eclipse Ti, Tokyo, Japan). Colour confocal images were constructed using NIS‐Elements C version 3.2 (Nikon Eclipse), and images were obtained with a 20× objective (Kim *et al*., 2012). For each experiment, two independent cultures were examined for at least 10 random positions. Biofilm formation was quantified by converting colour confocal images (20 image stacks) to grey scale using ImageJ, and COMSTAT biofilm software (Heydorn *et al*., 2000) was used to calculate biomasses (μm^3^ μm^−2^), mean biofilm thicknesses (μm) and substratum coverages (%). For each biofilm image, stack threshold was fixed and divided into four positions and 20 planar images per position were analysed.

### RNA isolation and quantitative real‐time PCR (qRT‐PCR)

For the qRT‐PCR assay, 25 ml of *C. albicans* at an initial turbidity of 0.1 at OD_600_ was inoculated into PDB broth in 250 ml Erlenmeyer flasks, followed by 4‐h incubation at 37°C with agitation (250 rpm) in the presence or absence of 7‐benzyloxyindole at 0.1 or 0.2 mM. To prevent RNA degradation, RNase inhibitor (RNAlater, Ambion, TX, USA) was added to cells. Total RNA was isolated using hot acidic phenol method (Amin‐ul Mannan *et al*., 2009), and RNA was purified using a Qiagen RNeasy mini Kit (Valencia, CA, USA). Expression of hyphae‐related genes (*ALS1, ALS3, ECE1, ECM38, EED1 EFG1, HYR1, HWP1, RBT1, SAP4* and *UME6*) was analysed. The specific primers and housekeeping gene (*RDN18*) used for qRT‐PCR are enlisted in Table S2. The qRT‐PCR method used has been previously described (Lee *et al*., 2011). SYBR Green master mix (Applied Biosystems, Foster City, CA, USA) and an ABI StepOne Real‐Time PCR System (Applied Biosystems) were used to perform qRT‐PCR. The assays were performed with at least two independent cultures.

### *Candida* infection in the *Caenorhabditis elegans* model

*Caenorhabditis elegans* strain N2 Bristol CF512 *fer‐15 (b26)*;* fem‐1 (hc17)* (Manoharan *et al*., 2017a,b) was used to perform *C. albicans* virulence assay using the protocol described by (Manoharan *et al*., 2017a,b). Briefly, synchronized adults worms were fed on *C. albicans* lawns for 4 h at 25°C and collected after washing three times with M9 buffer. Approximately 10 worms were then added to each well of 96‐well plates containing PDB medium (300 μl) with or without tested compounds at final concentrations of 0.02 or 0.05 mM. The assay plates were then incubated at 25°C for 4 days without shaking. For toxicity assays, 10 non‐infected worms were pipetted into single wells of a 96‐well dish containing M9 buffer and solutions of the compounds (200 μl) were added to final concentrations of 0.05, 0.1 or 0.5 mM. Plates were then incubated at 25◦C for 4 days without shaking. Three independent experiments were conducted in triplicate. Results were expressed as percentages of alive or dead worms as determined by their response to platinum wire touching after 4 days of incubation. Observations were made using an iRiS™ Digital Cell Imaging System (Logos Bio Systems).

### Statistical analysis

All the experiments were conducted, and results are expressed as means of two independent experiment values with standard deviation. The significant differences between treated and non‐treated samples were determined by Student's *t* test. *P* values < 0.05 were considered as statistical significance, and indicated by asterisks.

## Conflict of interest

None declared.

## Supporting information


**Table S1.** Effects of indole derivatives on *C. albicans* DAY185 biofilm formation.
**Table S2**. Sequences of the primers used for quantitative RT‐PCR.
