# Supplementary material for: Efficacy of 7‐benzyloxyindole and other halogenated indoles to inhibit Candida albicans biofilm and hyphal formation
Source: Microb Biotechnol. 2018 Apr 15;11(6):1060–9. doi: 10.1111/1751-7915.13268 (PMC6196399; doi:10.1111/1751-7915.13268)
Supplement: Supplementary file 1 — Table S1. Effects of indole derivatives on C. albicans DAY185 biofilm formation. Table S2. Sequences of the primers used for quantitative RT‐PCR. [file MBT2-11-1060-s001.docx]

**Table S1. Effects of indole derivatives on *C. albicans* DAY185 biofilm formation.** Biofilm screenings of the effects of indole derivatives on *C. albicans* DAY185 were performed after incubation in PDB at 37ºC for 24 h in 96-well plates. Percentage ratios represent biofilm inhibition versus that of untreated controls. Percentage ratio of biofilm inhibition >70% are shown in bold.

| **Name** | **Biofilm inhibition (%)** | | **Name** | **Biofilm inhibition (%)** | |
| --- | --- | --- | --- | --- | --- |
|  | **0.5 mM** | **0.1 mM** |  | **0.5 mM** | **0.1 mM** |
| 7-Azaindole | 14±24 | ND | Indole | 30±2 | 5±9 |
| 5-Bromo-3-iodo-7-azaindole | 6±11 | ND | Indole-3-acetonitrile | 42±18 | ND |
| **7-Bromoindole** | **96±12** | 10±25 | **Indole-3-acetic acid** | **70±24** | 3±12 |
| **4-Benzyloxyindole** | **95±1** | **75±4** | Indole-3-acetamide | 9±14 | ND |
| **5-Benzyloxyindole** | **98±4** | **94±3** | **Indole-3-butyric acid** | **98±1** | 54±14 |
| **6-Benzyloxyindole** | **80±4** | **70±3** | Indole-3-carbinol | 11±13 | ND |
| **7-Benzyloxyindole** | **96±4** | **94±3** | Indole-3-carboxyaldehyde | 50±28 | ND |
| 7-Chloroindole | 50±15 | ND | Indole-7-carboxylic acid | 46±16 | ND |
| 5,6-Difluoroisatin | 7.5±16 | ND | **Indole-3-propionic acid** | **95±3** | 8±17 |
| 4**-Fluoroindole** | **99±1** | **92±7** | **5-Iodoindole** | **98±2** | **75±11** |
| 5-Fluoroindole | 16±6 | ND | **Isatin** | **80±20** | 2±12 |
| **6-Fluoroindole** | **97±4** | 26±8 | 3,3'-Methylene bisindole | 5.5±18 | ND |
| 7-Fluoroindole | 30±3 | ND | 7-Methoxyindole | 5.7±34 | ND |
| 5-Fluoroxiindole | 42±15 | ND | **5-Nitroisatin** | **79±17** | 4±13 |
| 5-Fluoroindolin-2,3-dione | 19±16 | ND | 7-Nitroindole | 11±13 | ND |
| 7-Fluoroindolin-2,3-dione | 2.9±13 | ND | 2-Oxindole | 10±17 | ND |
| 8-Fluoroquinoline | 18±14 | ND | **5-(Trifluoromethoxy)**  **indoline-2,3-dione** | **97±3** | 5±12 |
| **7-Formylindole** | **91±6** | 6±11 | 7-(Trifluoromethyl)  indoline-2,3-dione | 3.9±19 | ND |
| 7-Hydroxyindole | 46±10 | ND |  |  |  |

ND, Not Done

**Supplementary Table 2.** Sequences of the primers used for quantitative RT-PCR

| **Group** | **Gene** | **Function** | **Primer (5’-3’)** | **References** |
| --- | --- | --- | --- | --- |
| House-keeping | *RDN18* | Structural constituent of ribosome/Translation | Forward - AGAAACGGCTACCACATCCCA | (Li *et al.*, 2012) |
|  |  |  | Reverse - CGAATGGGCCCTGTATCGT |  |
| Hypha-specific | *ALS1* | Agglutinin-like protein 1 | Forward - AGCTGTTGCCAGTGCTTC  Reverse - AATGTGTTGGTTGAAGGTGAG | (Hsu *et al.*, 2013) |
|  | *ALS3* | Agglutinin-like protein 3 | Forward - CAACATCAACCAACCAATCTC  Reverse - TGAATAACAGAACCAGATCCG | (Tsang *et al.*, 2012) |
|  | *EFG1* | Enhanced filamentous growth protein (Positive regulator for *ECE1*, *ECE2* and *ALS3*) | Forward – TATGCCCCAGCAAACAACTG  Reverse - TTGTTGTCCTGCTGTCTGTC | (Tsang *et al.*, 2012) |
|  | *HYR1* | Hyphally regulated protein | Forward - TTGTTTGCGTCATCAAGACTTTG  Reverse - GTCTTCATCAGCAGTAACACAACCA | (Tsang *et al.*, 2012) |
| Biofilm/  adhesion | *ECE1* | Extent of cell elongation  protein | Forward - CCAGAAATTGTTGCTCGTGTTGCCA  Reverse - TCCAGGACGCCATCAAAAACGTTAG | (Manoharan *et al.*, 2017)) |
|  | *ECM38* | Extra cellular mutant | Forward - GCGGTTTCTGCTGCTTTAACAA  Reverse - ACCAGATCCATTTAACCCCAACAC | This study |
|  | *EED1* | Epithelial escape and dissemination | Forward - AGCAACGACTTCCAAAAGGA | (Hsu *et al.*, 2013) |
|  |  |  | Reverse - CGGTTTCTGGTTCGATGATT |  |
|  | *HWP1* | Hyphal cell wall protein | Forward - TGGTGCTATTACTATTCCGG | (Sun *et al.*, 2015) |
|  |  |  | Reverse - CAATAATAGCAGCACCGAAG |  |
|  | *RBT1* | HWP1 homolog | Forward- CTGCCATTCAACCATCTGCTAACTCCTCATAC | (Manoharan *et al.*, 2017) |
|  |  |  | Reverse- CAGCAAGACCAATAATAGCAGCACCATAAGT |  |
|  | *SAP4* | Secreted aspartyl proteinase4 | Forward - GGTACCGTTGATTTCCAATTC | (Tsang et al., 2012) |
|  |  |  | Reverse - ATCTTCACTTTCACGAACACG |  |
|  | *UME6* | Unscheduled meiotic gene expression | Forward - AGCACCAAATTCGCCTTATG  Reverse - AGGTTGAGCTTGCTGCAGTT | (Hsu *et al.*, 2013) |

**Reference**

Hsu, C.C., Lai, W.L., Chuang, K.C., Lee, M.H., and Tsai, Y.C. (2013) The inhibitory activity of linalool against the filamentous growth and biofilm formation in Candida albicans, *Med Mycol* **51**: 473-482.

Li, Q.Q., Skinner, J., and Bennett, J.E. (2012) Evaluation of reference genes for real-time quantitative PCR studies in *Candida glabrata* following azole treatment, *BMC Mol Biol* **13**: 22.

Manoharan, R.K., Lee, J.-H., Kim, Y.-G., and Lee, J. (2017) Alizarin and chrysazin inhibit biofilm and hyphal formation by *Candida albicans*, *Front Cell Infect Microbiol* **7**: 447.

Sun, L., Liao, K., and Wang, D. (2015) Effects of magnolol and honokiol on adhesion, yeast-hyphal transition, and formation of biofilm by *Candida albicans*, *PLoS One* **10**: e0117695.

Tsang, P.W., Bandara, H.M., and Fong, W.P. (2012) Purpurin suppresses *Candida albicans* biofilm formation and hyphal development, *PLoS One* **7**: e50866.
